# Biological characteristics and metabolic profile of canine mesenchymal stem cells isolated from adipose tissue and umbilical cord matrix

**DOI:** 10.1371/journal.pone.0247567

**Published:** 2021-03-04

**Authors:** Romina Marcoccia, Salvatore Nesci, Barbara Merlo, Giulia Ballotta, Cristina Algieri, Alessandra Pagliarani, Eleonora Iacono

**Affiliations:** 1 Department of Veterinary Medical Sciences, University of Bologna, Ozzano Emilia, Bologna, Italy; 2 Health Science and Technologies Interdepartmental Center for Industrial Research (HST-ICIR), University of Bologna, Bologna, Italy; Faculty of Animal Sciences and Food Engineering, University of São Paulo, BRAZIL

## Abstract

Despite the increasing demand of cellular therapies for dogs, little is known on the differences between adult and fetal adnexa canine mesenchymal stem cells (MSCs), and data on their metabolic features are lacking. The present study aimed at comparing the characteristics of canine adipose tissue (AT) and umbilical cord matrix (UC) MSCs. Moreover, for the first time in the dog, the cellular bioenergetics were investigated by evaluating the two main metabolic pathways (oxidative phosphorylation and glycolysis) of ATP production. Frozen-thawed samples were used for this study. No differences in mean cell proliferation were found (P>0.05). However, while AT-MSCs showed a progressive increase in doubling time over passages, UC-MSCs showed an initial post freezing-thawing latency. No differences in migration, spheroid formation ability, and differentiation potential were found (P>0.05). RT-PCR analysis confirmed the expression of CD90 and CD44, the lack of CD14 and weak expression of CD34, mostly by AT-MSCs. DLA-DRA1 and DLA-DQA1 were weakly expressed only at passage 0 by UC-MSCs, while they were expressed at different passages for AT-MSCs. There was no difference (P>0.05) in total ATP production between cell cultures, but the ratio between the “mitochondrial ATP Production Rate” and the “glycolytic ATP Production Rate” was higher (P<0.05) in AT- than in UC-MSCs. However, in both MSCs types the mitochondrial respiration was the main pathway of ATP production. Mitochondrial respiration and ATP turnover in UC-MSCs were higher (P<0.05) than in AT-MSCs, but both had a 100% coupling efficiency. These features and the possibility of increasing the oxygen consumption by a spare respiratory capacity of four (AT-MSCSs) and two (UC-MSCs) order of magnitude greater than basal respiration, can be taken as indicative of the cell propensity to differentiate. The findings may efficiently contribute to select the most appropriate MSCs, culture and experimental conditions for transplantation experiments in mesenchymal stem cell therapy for companion animals.

## Introduction

Stem cells are unspecialized cells with the ability to renew themselves for long periods without changes in their properties [1]. Mesenchymal stem cells (MSCs) are one type of non-hemopoietic stem cells, originating from mesoderm and present in different types of tissues [2, 3]. Furthermore, MSCs possess multi lineage differentiation capacity into chondrocytes, osteocytes, adipocytes and other cell types like myoblasts, bone marrow stromal cells, fibroblasts, cells co creating connective tissue of the body, as well as ligaments and tendons [4, 5]. Mesenchymal stem cells are characterized by the expression of typical mesenchymal surface Cluster Designation (CD), such as CD105, CD73 and CD90 and lack of hematopoietic ones, CD45, CD34, CD14 or CD11b, CD79α or CD19 and HLA-DR [2].

Due to their properties, MSCs offer a great chance for cell-based therapies and tissue engineering applications in veterinary medicine, especially for canines (*Canis familiaris)*. The effective management of companion animals, which often suffer from age-related diseases such as arthrosis and degenerative diseases due to their increased longevity, requires sophisticated new treatments and preventive strategies. Moreover, dogs could be employed as a model for human genetic and acquired diseases, helping to define the potential therapeutic efficiency and safety of stem cell therapy [6, 7].

To date, canine derived MSCs have been isolated from different adult tissues, such as liver, bone marrow [8], and adipose tissue [9, 10]. Adipose tissue (AT) is easily available and contains a high number of MSCs, especially in the perivascular region, which makes it a valid source of stem cells for autologous or heterologous use [11]. In canine species, the clinical use of AT-MSCs is versatile thanks to their differentiation potential and secretion of numerous immunomodulatory factors [12]. Nevertheless, MSCs derived from fetal adnexa, such as umbilical cord (UC) matrix, which are discarded at parturition, could overcome many of the limitations of adult tissues-derived MSCs, such as the invasive procedure required for sample recovery [13, 14]. Thanks to its extracorporeal nature, these cells are therefore easy to obtain non-invasively and without pain or stress for the donor [15, 16].

The metabolism gathers tuned biochemical pathways for maintenance of cellular homeostasis and cellular self-renewal capability [17]. To adequately select and address MSC therapeutic applications, a first task is to understand the biochemical and metabolic features of these cells. Data on MSCs metabolism are still controversial: stem cells prefer glycolysis to mitochondrial respiration when located in the niche because the environment could be hypoxic [17] and glycolysis is required by macromolecule and biostructure synthesis [18]. Despite some studies comparing canine adult and fetal adnexa derived MSC growth and differentiation capacity [19, 20], as far as we are aware no data on the metabolic profile are available. The aim of this study was to compare, for the first time, the metabolic profile of canine MSCs derived from adipose and umbilical cord tissues. The metabolic and bioenergetic profiles complement to selected biological features, such as cellular proliferation, differentiation potential, molecular profile, migration and spheroid formation ability, to provide an overall pattern of the characteristics of these cells as first step for their potential future use in therapy.

## Materials and methods

Chemicals were obtained from Sigma Aldrich (Merck); type I collagenase, DMEM (Dulbecco’s Modified Eagle’s Medium) high glucose medium with Glutamax, MEM (Modified Eagle’s Medium) with Glutamax, and Fetal Bovine Serum (FBS) are branded GIBCO (ThermoFisher Scientific).

Laboratory plastic ware was from Falcon (VWR), unless otherwise stated.

### Animals

Intra-abdominal fat tissue from healthy one-year old dogs (n = 4) was recovered at castration, while umbilical cord samples (n = 4) were recovered at ovary-hysterectomy of a 30 days pregnant bitch. All animals were referred to the Department of Veterinary Medical Sciences (University of Bologna), and the written consent was given by the owners to allow the use of removed tissue for research purposes. Experimental procedures were approved by the Ethics Committee on animal use of the University of Bologna (Prot. 55948-X/10), and by the Italian Ministry of Health.

### Sample collection and cell isolation

Immediately after removal, AT and UC samples were stored in DPBS (Dulbecco’s Phosphate Buffered Saline) supplemented with antibiotics (100 IU/mL penicillin, 100 μg/mL streptomycin) at 4°C until the transfer to the lab. The whole portion of placental UC, freed from blood vessels, was used. MSCs were isolated as previously described by Iacono et al. [10]. Briefly, under a laminar flow hood, tissue was rinsed by repeated immersion in DPBS, weighed and finely minced (0.5 cm) using sterile scissors. Minced tissue was transferred into a 50 mL polypropylene tube and a digestion solution, containing 0.1% collagenase type I dissolved in DPBS, was added (1 mL solution/1 g tissue) mixing thoroughly. This mix was kept in a 37 °C water bath for 30 min and mixed vigorously every 10 min. After incubation the collagenase was inactivated, diluting 1:1 with DPBS supplemented with 10% FBS and the resulting solution was filtered in order to discard the undigested tissue. Nucleated cells were pelleted at 470 *g* for 10 min at 25°C. The obtained pellet was suspended in culture medium composed of DMEM/MEM 1:1, plus 10% FBS added with antibiotics (100 IU/mL penicillin, 100 μg/mL streptomycin). Cells were plated in a 25 cm^2^ flask in 5 mL of culture medium and the flask was marked as “Passage 0” (P0). Cells were cultured in humidified air with 5% CO_2_ at 38.5°C. After 48h of *in vitro* culture, the medium was completely re-placed and non-adherent cells removed. Medium was then changed twice a week until 80 to 90% confluence was reached.

### Cell freezing and thawing

Since samples were recovered in different moments, isolated P0 cells were frozen and stored in liquid nitrogen in order to perform all tests at the same time. AT- and UC-MSCs were deep-frozen as previously described by Merlo et al. [21]. Briefly, cells were allowed to proliferate until 80 to 90% confluence, thereafter they were dissociated by 0.25% trypsin for 10 min. Trypsin was then inactivated diluting 1:2 with DPBS+10% FBS. MSCs were centrifuged at 470g for 10 min at 25°C. The pellet was suspended in 0.5 mL of FBS, transferred in a 1.5 mL cryogenic tube (Sarstetd Inc.) and put at 5 to 8° C for 10 min. At the end of this period, the cells were diluted 1:1 with FBS+16% DMSO (dimethyl sulfoxide) for a final concentration of 8% DMSO. After other 10 min at 5 to 8° C, the cryogenic tube was put at -80°C for 24h in Mr Frosty (Nalgene) and finally stored in liquid nitrogen. AT- and UC-MSCs were thawed at 37°C and diluted with 20 mL culture medium, then centrifuged at 470 *g* for 10 min at 25°C. The pellet was suspended in 1 mL culture medium and the cells concentration was evaluated by using a haemocytometer. Cells were plated in a 25 cm^2^ flask (5000 cells/cm^2^) as “Passage 1” (P1). Cells were allowed to proliferate until 80 to 90% confluence before trypsinization and successive passage.

### Cell culture and population doublings

Calculation of cell-doubling time (DT) and cell-doubling number (CDN), that is the approximate number of doublings that the cell population has undergone since isolation, was carried out according to the following formulae [22]:

CDN=ln(Nf/Ni)/ln (1)

where N_f_ and N_i_ are the final and the initial number of cells, respectively;

DT=CT/CDN

where CT is the cell culture time.

The experiment was done in three replicates.

### CFU (Colony forming unit) assay

To assess colony formation ability, 1x10^4^ cells at P1 were seeded in a 90 mm petri dish and cultured for 13 days [23]. For each sample three replicates were carried out. Colonies were fixed in 4% paraformaldehyde at room temperature (RT) and stained for 15 min with Giemsa 0,1% stain. Colonies formed by at least 16–20 nucleate cells were counted using an inverted light microscope (Eclipse TE 2000u, Nikon).

### Spheroid formation and migration assays

To define differences between AT- and UC-MSCs, spheroid formation and migration tests were performed. Each test was replicated 3 times for all samples.

For spheroid formation assay, P3 cells were cultured in multiwell Corning 96-well Black/Clear Round Bottom Ultra- Low Attachment Spheroid Microplate (5000 cells/25 μL drop) for 24 h. This method provides information about the direct cell-cell adhesion architecture found in normal tissues, differently from the cell-substratum adhesion, performed on monolayer cultures adherent to rigid substrates. Images were acquired by a CCD camera (DS-Fi2, Nikon) mounted on a light inverted microscope. Starting from the binary masks obtained by Image J software (imagej.nih.gov/ij/), the volume of each spheroid was computed using ReViSP (sourceforge.net/projects/revisp) [24], a software specifically designed to accurately estimate the volume of spheroids and to render an image of their 3D surface.

To assess cell migration potential, wound-healing assay was carried out, as previously described by Liang et al. [25]. Briefly, P3 cells were plated (5000 cell/cm^2^) in a 35 mm petri dish. At 80 to 90% confluence the cell monolayer was scraped using a 1000 μL pipet tip. After washing twice with DPBS, the dish was incubated for 24 h. Images were acquired both immediately after the tip-scratch (time 0 = T0) and after the incubation period (time 1 = T1), and the distances of each scratch closure were calculated by ImageJ software (imagej.nih.gov/ij/). The migration percentages were calculated using the following formula [26]:

[(distanceatT0-distanceatT1)*100]/distanceatT0

### Multi lineage differentiation

*In vitro* differentiation potential toward osteogenic, adipogenic and chondrogenic lineages of AT- and UC-MSCs were studied. Cells (5000 cells/cm^2^) were cultured for three weeks under specific induction media (Table 1) [27]. As negative control, an equal number of cells was cultured in expansion medium. The specific induction media and expansion medium were replaced twice a week. The *in vitro* differentiation potential was assessed at P3 of the *in vitro* culture. Three replicates for each sample were carried out. For the cytological evaluation of the differentiation, cells were fixed with 4% paraformaldehyde at RT for 1 h and then stained with Oil Red O, Alcian Blue and Alizarin Red for adipogenic, chondrogenic and osteogenic induction, respectively. Stained cells were observed under an inverted light microscope and photographed. Images obtained after chondrogenic and osteogenic differentiation were analysed for colour intensity using Image J software [28]. The intensity of adipogenic differentiation was assessed using a scoring system based on Oil Red O staining (Table 2) [29].

**Table 1:** Specific induction media compositions.

Adipogenic medium	Chondrogenic Medium	Osteogenic Medium
• DMEM/MEM	• DMEM/MEM	• DMEM/MEM
• 10% FBS	• 1% FBS	• 10% FBS
• 0.5 mM IBMX	• 6.25 μg/mL insulin	• 50 μM AA2P
(removed after 3 days)	• 50 nM AA2P	• 0.1 μM DXM
• 1 μM DXM	• 0.1 *μ*M DXM	• 10 mM BGP
(removed after 6 days)	• 10 ng/mL hTGF-β1	
• 10 μg/mL insulin		
• 0.1 mM indomethacin		

IBMX: isobutylmethylxanthine, DXM: Dexamethasone, hTGF: human Transforming Growth Factor, AA2P: Ascorbic Acid 2-Phosphate, BGP: Beta-Glycerophosphate

**Table 2:** Semi-quantitative scoring system used in the evaluation of adipogenic differentiation.

Score	% of differentiated cells	Size and arrangement of lipid droplets
0	0–5	No droplets
1	>5–50	Predominantly isolated and small
2	>50–80	Predominantly medium-sized
3	>80–100	Predominantly large

### Molecular characterization

Expression of specific mesenchymal (CD44, CD90), hematopoietic (CD34, CD14), and major histocompatibility complex (DLA-DQA1, DLA-DRA1) markers was investigated by RT-PCR analysis in undifferentiated canine AT- and UC-MSCs. The specific set of primers used are listed in Table 3. All tests were performed at each passage of *in vitro* culture on 10^5^ cells, previously snap-frozen at -80°C. The RNA was extracted using Nucleo Spin RNA kit (Macherey-Nagel, Düren, Germany) following the manufacturer’s instructions. cDNAs were synthesized by RevertAid RT Kit (ThermoFisher Scientific, Waltham, Massachusetts, USA) and used directly in PCR reactions, following the instructions of Maxima Hot Start PCR Master Mix (2X) (ThermoFisher Scientific, Waltham, Massachusetts, USA). Canine glyceraldehyde-3-phosphate dehydrogenase (GAPDH) was employed as reference gene for each sample in order to standardize the results and to assess RNA purity. For all primers, in order to assess sample purity, a negative RT and a mix without primer were analyzed. PCR products were visualized (Benchtop 2UV/PhotoDoc-It 65, UVP International PBI) on an ethidium bromide stained 2% agarose gel.

**Table 3:** Sequences of primers used for RT-PCR analysis.

Gene	Primer sequences (5′→3′)	Amplicon (bp)	Reference
**Reference gene**			
**GAPDH**	FW: GGTCACCAGGGCTGCTTT	209	[30]
	RV: ATTTGATGTTGGCGGGAT		
**Mesenchymal markers**			
**CD90**	FW: CAGCATGACCCGGGAGAAAAAG	134	[31]
	RV: TGGTGGTGAAGCCGGATAAGTAGA		
**CD44**	FW: GCCCTGAGCGTGGGCTTTGA	268	[15]
	RV: TCTGGCTGTAGCGGGTGCCA		
**Hematopoietic markers**			
**CD14**	FW: CCCGGCGCTCACCACCTTAGAC	98	[31]
	RV: CCTGGAGGGCCGGGAACTTTTG		
**CD34**	FW: GCCTGCTCAGTCTGCTGCCC	255	[15]
	RV: TGGTCCCAGGCGTTAGGGTGA		
**Major histocompatibility complex markers**			
**DLA-DRA1**	FW: CGCTCCAACCACACCCCGAA	246	[15]
	RV: GGCTGAGGGCAGGAAGGGGA		
**DLA-DQA1**	FW: GCACTGGGGCCTGGATGAGC	163	[15]
	RV: ACCTGAGCGCAGGCCTTGGA		

### Cellular bioenergetics

The Seahorse XFp analyzer (Agilent) was used to simultaneously measure oxygen consumption rate (OCR), an index of cell respiration (pmoL/min), and extracellular acidification rate (ECAR), an index of glycolysis (mpH/min). AT- and UC-MSCs were trypsinized and grown in XFp cell culture miniplates (Agilent) at 10^4^ cells per well one day prior to the assay. On the day of an experiment, MSCs were switched to freshly made Seahorse XF DMEM medium pH 7.4 supplied with 10 mM glucose, 1 mM sodium pyruvate, and 2 mM L-glutamine. The plates were incubated at 37°C in air for 45 minutes before measuring OCR and ECAR by the adequate programs (ATP Rate Assay or Cell Mito Stress Test). The injection ports of XFp sensor cartridges, which were hydrated overnight with XF calibrant at 37°C, were loaded with 10x concentration of inhibitors according to the instructions provided by Seahorse XFp ATP Rate Assay and CellMito Stress Test. The final concentration used for ATP Rate Assay were 1.5 μM oligomycin (port A) and 0.5 μM rotenone plus antimycin A (port B), while for Cell Mito Stress Test the final concentrations were 1.5 μM oligomycin (port A), 2.0 μM and 0.5 μM FCCP (port B) with AT- and UC-MSCs, respectively and 0.5 μM rotenone plus antimycin A (port C). All the analysis were run at 37°C. All data were analyzed by WAVE software version 2.6.1; OCR and ECAR values were normalized to the total number of cells for each well. All parameter values were calculated per well according to the manufacturer’s instructions. Both ATP Rate Assay and Mito Stress Test were carried out three times on different days for each cell type.

The ATP Rate Assay provides the bioenergetic parameters currently used to characterize cellular ATP production, namely ATP production rate, related to the conversion of glucose to lactate in the glycolytic pathway (glycoATP Production Rate) and to mitochondrial oxidative phosphorylation (mitoATP Production Rate). Accordingly, the ratio between mitoATP Production Rate and glycoATP Production Rate (ATP Production Rate Index) is currently considered as a valuable parameter to detect changes and/or differences in the metabolic phenotype (ratio > 1 mainly OXPHOS pathway; ratio < 1 mainly glycolytic pathway). The Mito Stress Test enables to characterize cell respiration by the following parameters: basal respiration, detected as baseline OCR before oligomycin addition; minimal respiration measured as OCR in the presence of oligomycin; maximal respiration evaluated as OCR after FCCP addition. The non-mitochondrial respiration, evaluated as OCR in the presence of rotenone plus antimycin A, was subtracted from all the above parameters. The ATP turnover or oligomycin-sensitive respiration was obtained from the difference between the basal respiration and the minimal respiration (OCR in presence of oligomycin). Finally, the difference between the maximal and the basal respiration provided the spare capacity, which represents the ability to respond to an increased energy demand and can be considered as a measure of the flexibility of the oxidative phosphorylation (OXPHOS) machinery.

### Statistical analysis

Cell-doubling number, cell-doubling time, percentages of migration and metabolic data are expressed as mean ± SD. Statistical analyses were performed using IBM SPSS Statistics 25 (IBM Corporation). Data were analyzed for normal distribution, using a Shapiro Wilk test. Mean DTs, CDs, number of colonies and migration rate were analyzed using Student’s T Test for unpaired variables, while the 3D spheroid volumes were compared using Mann-Whitney U-test. Metabolic data were analyzed by One-way ANOVA, followed by Students-Newman-Keuls’ test when *F* values indicated significance. Significance was assessed for P<0.05.

## Results

### Cell proliferation

Adherent cells with the characteristic spindle-shaped morphology were isolated from all samples of adipose tissue and umbilical cord matrix. For all samples of both tissues, undifferentiated cells were cultured until P4 after freezing-thawing, without changes in cell morphology. No statistically significant differences were observed in mean CDN (AT-MSCs 10.3±1.9 *vs* UC-MSCs 11.5±0.8; P>0.05). Furthermore, mean DTs were similar (P>0.05) (AT-MSCs 85.1±43.8 h *vs* UC-MSCs 83.2±18.5h).

**Fig 1:**
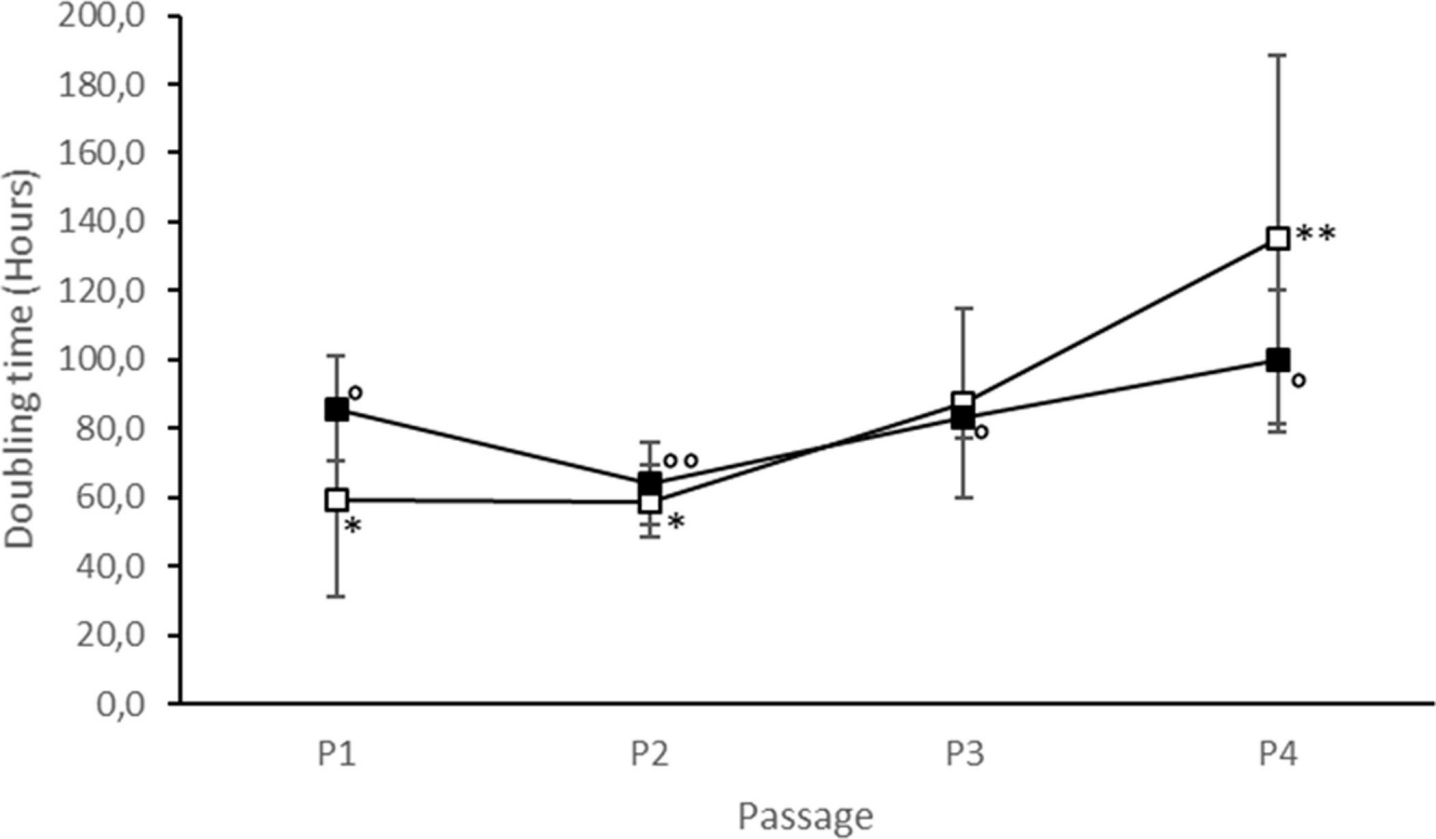
Cell growth of canine AT- and UC-MSCs. Doubling times of frozen-thawed AT-MSCs (□) and UC-MSCs (■) over four passages of *in vitro* culture. Data represent the mean ± SD. Different symbols indicates significant differences: * and ** for AT-MSCs, ° and °° for UC-MSCs (P <0.05).

Comparing data among passages of the same cell culture, AT-MSCs showed a progressive increase in DTs over time. DT at P4 (134.9±53.7 h) was significantly higher (P<0.05) than at P1 (59.4±28.2 h) and P2 (58.7±10.5 h) (Fig 1). On the other hand, UC-MSCs showed a slightly different trend (Fig 1). DT of UC-MSCs at P1 (85.7±15 h) was significantly higher (P<0.05) than DT at P2 (64.0±12.8 h) and similar (P>0.05) to DT at P3 (83.3±6.0 h) and P4 (99.7±20.8 h), while DT at P2 (64.0±12.8 h) was the one significantly lower (P<0.05) than those registered at subsequent passages.

The mean number of colonies formed at P1 by AT-MSCs (87,8±51,8) and UC-MSCs (41,0±35,4) was not significantly different (P>0.05).

### Spheroid formation and migration assays

**Fig 2:**
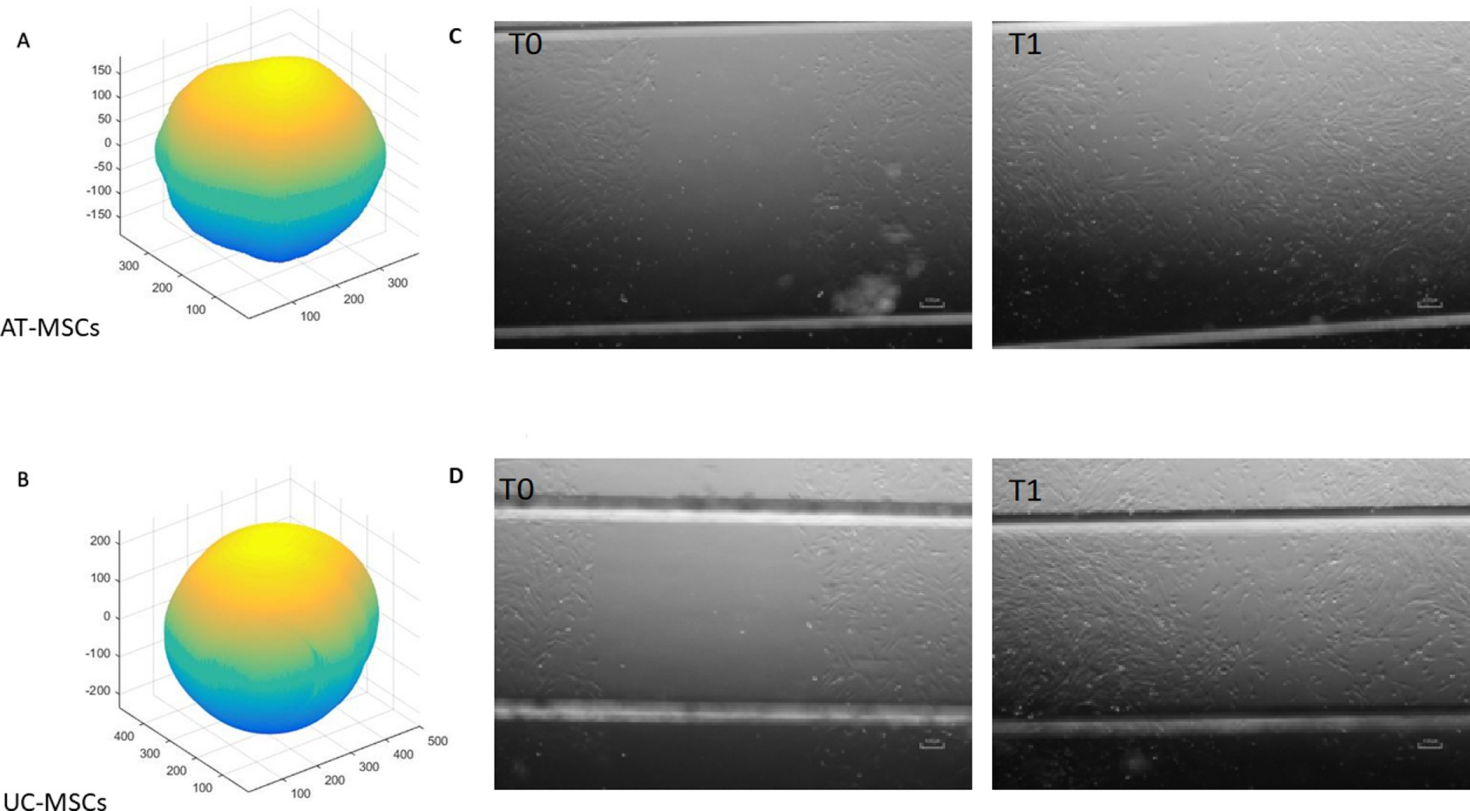
Spheroid formation and scratch assays of canine AT- and UC-MSCs. A-B) Volume reconstruction of an AT-MSC (A) and UC-MSCs (B) spheroid, obtained after 24 h of hanging drop culture. C-D) Scratch assay on AT-MSCs (C) and UC-MSCs (D) at T0 and after 24 h (T1) of cell growth (Magnification 4X).

All samples of AT- and UC-MSCs, cultured in specific multi-well plate, were able to form spheroids. The volume of formed spheroids was similar between AT- and UC-MSCs (P>0.05) (Fig 2A–2B), confirming a comparable cell-cell adhesion ability. Furthermore, no differences were registered in the migration ability between the two groups (AT-MSCs 53.5±11.8% *vs* UC-MSCs 49.0±24.2%; P>0.05), as demonstrated by the cell distribution (Fig 2C–2D).

### Multilineage differentiation

**Fig 3:**
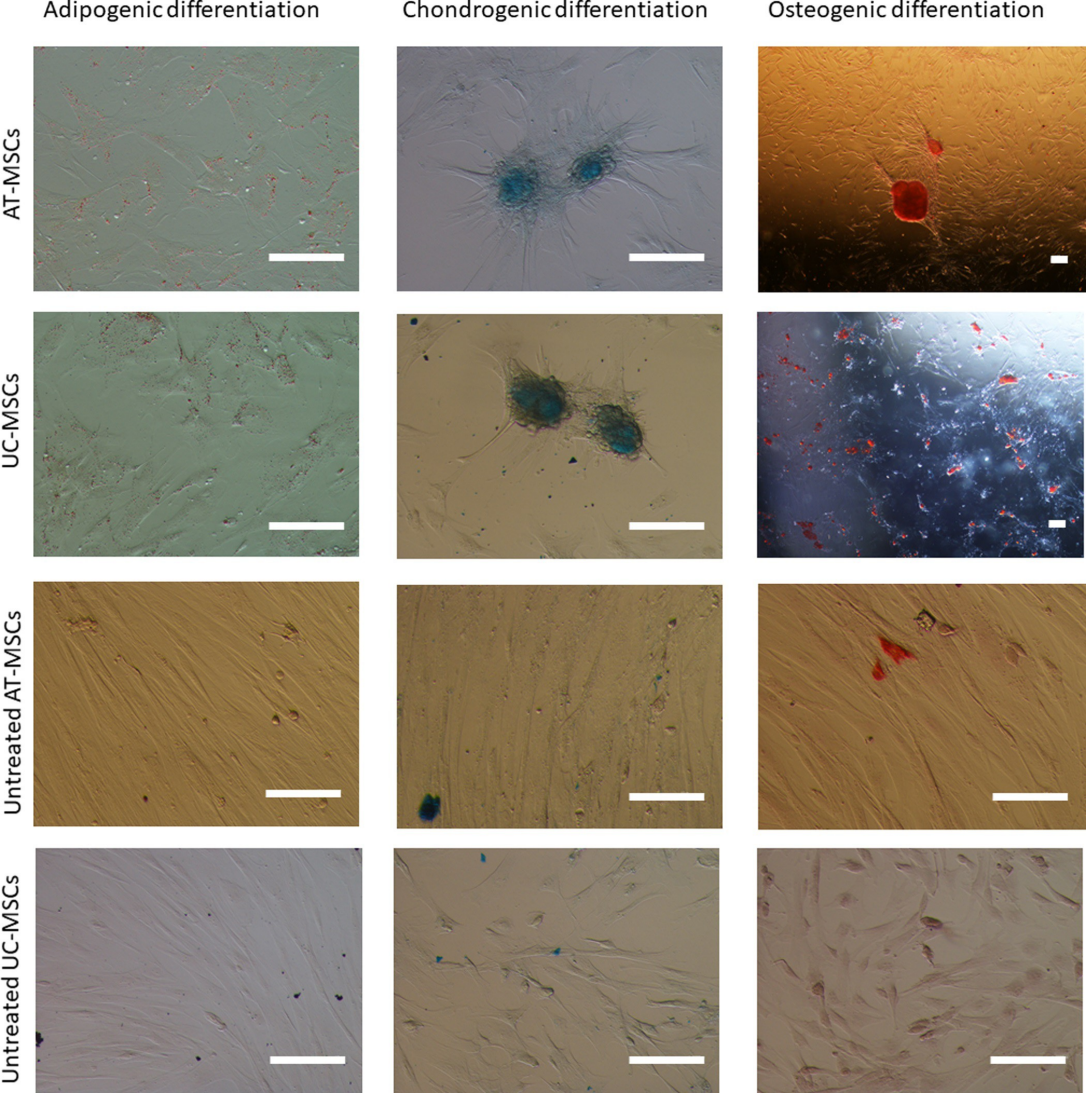
Differentiation potential of AT- and UC-MSCs. Canine AT- and UC-MSCs cultured toward adipogenic, chondrogenic and osteogenic differentiation. Magnification: 20X for all pictures except for 4X of osteogenic differentiated cells (bars = 100 μm).

After adipogenic differentiation, the changes in the two types of MSCs were similar, and the original typical fibrous cell shape changed in shortened and rounded ones. Lipid droplets were observed in the cytoplasm of AT- and UC-MSCs. After Oil red O staining, the lipid droplets were stained red (Fig 3). The two groups reached a score of 2 (showing >50–80% positive cells with medium-size droplets).

After three weeks of osteogenic induction, cell morphology changed from fibers to polygons and scales. Calcium nodules appeared in both AT- and UC-MSCs. All MSCs showed red mineralized deposits. Observing cells at low magnification (4X) (Fig 3) AT-MSCs formed less numerous but larger deposits, while UC-MSCs tended to form more numerous but smaller deposits. These different features lead to a similar colour intensity between the two cell types (P>0.05).

Chondrogenic differentiation was observed after 21 days in MSCs from both groups (AT and UC); the cell shape changed and matrix secretion was observed. Chondrogenic potential was confirmed by Alcian Blue (Fig 3), which enabled the identification of an extracellular matrix rich in proteoglycans. Also for chondrogenic differentiation, the colour intensity was similar for the two MSC cultures (P>0.05).

### Molecular characterization by RT-PCR

**Fig 4:**
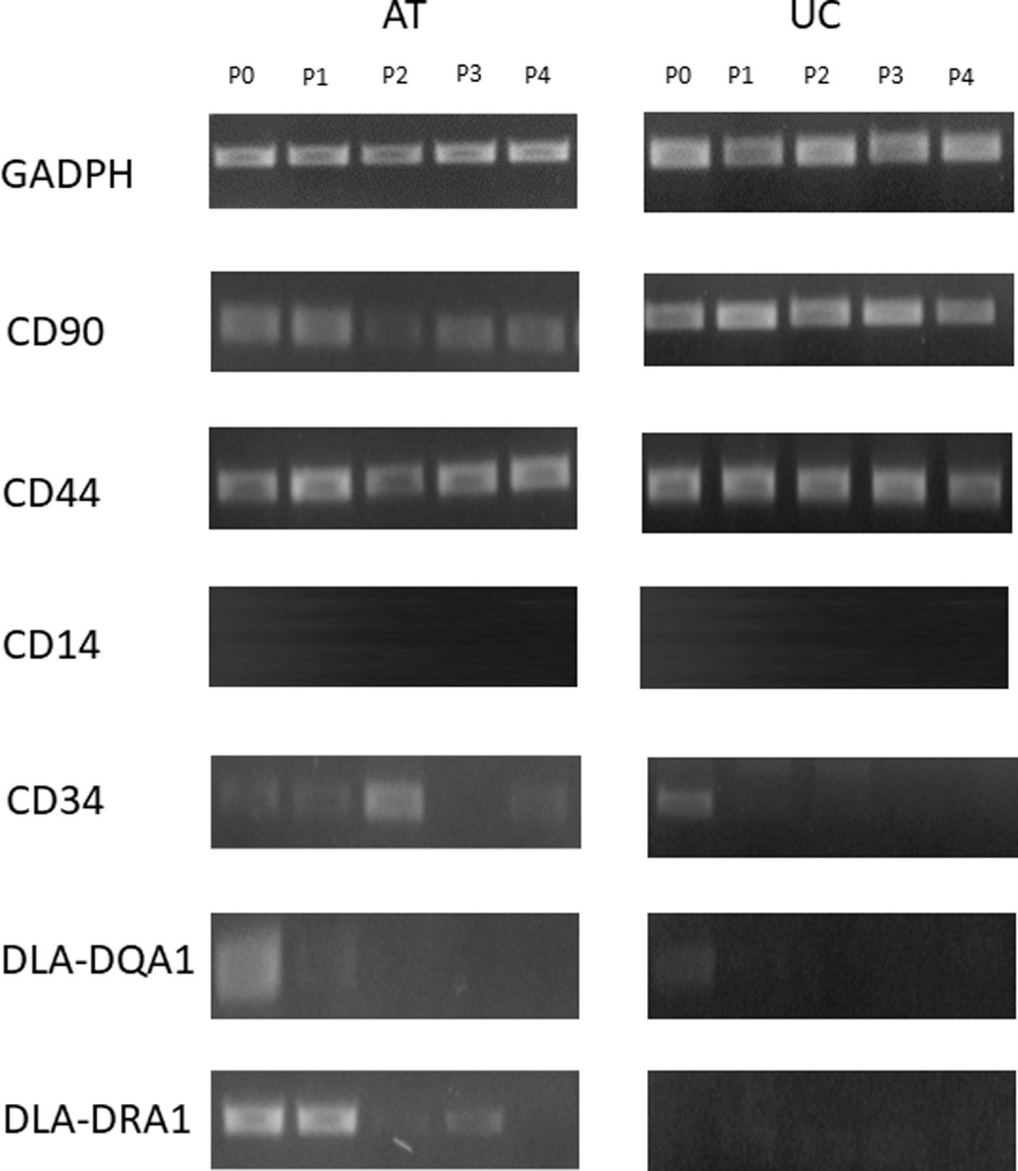
RT-PCR analysis of gene expression in canine AT- and UC-MSCs. Products from AT- and UC-MSCs samples are visualized over time, from passage 0 (P0) to passage 4 (P4) of *in vitro* culture.

Data is shown in Fig 4. All samples of AT- and UC-MSCs expressed typical mesenchymal markers, CD90 and CD44, at every passage of the *in vitro* culture. No samples expressed the typical hematopoietic marker CD14. The hematopoietic marker CD34 was expressed in different manner by AT- and UC-MSCs. In fact, an individual variability between AT samples was observed, since CD34 was poorly expressed at passage P3 by one adipose tissue sample, while it was weakly expressed at all passages (P0-P4) by the other two samples. The same marker, instead, was weakly expressed only at P0 by UC-MSCs in all samples. As for CD34, an individual variability among adipose tissue samples was observed in the expression of DLA-DQA1 and DLA-DRA1, while they were weakly expressed only at P0 by UC-MSCs.

### Cellular ATP production

**Fig 5:**
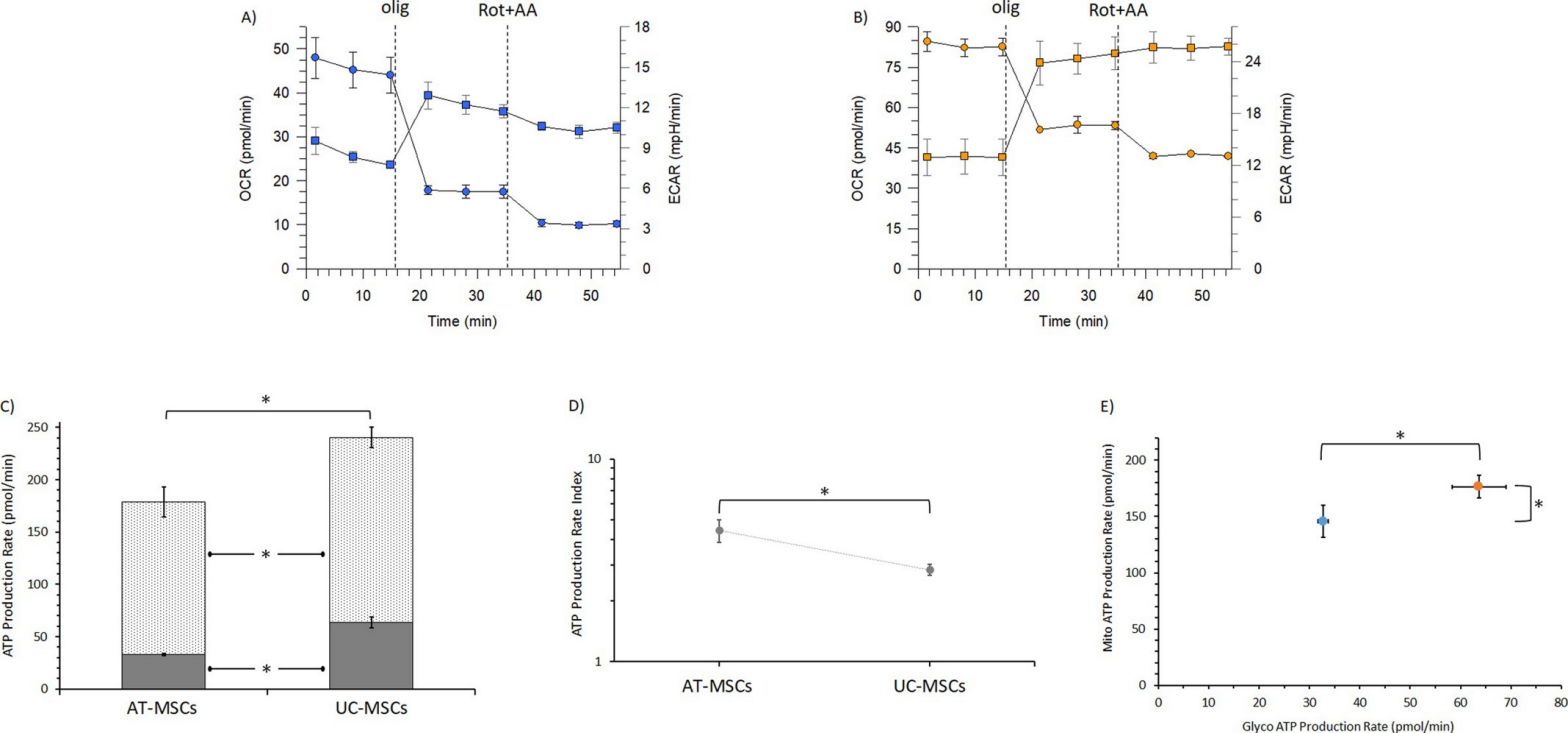
Real-Time ATP Production Rate Assay of basal OCR (blue spheres) and ECAR (blue squares) rates of AT-MSCs (A) and OCR (orange spheres) and ECAR (orange squares) rates of UC-MSCs (B). After detection of OCR and ECAR rates, oligomycin (1.5 μM) and rotenone plus antimycin A (0.5 μM) were serially injected at fixed times in order to allow to detect the mitochondrial and glycolytic ATP production rates. C) AT- and UC-MSCs quantification of ATP production by mitochondrial oxidative phosphorylation (dashed rectangle) or by the glycolytic pathway (glucose conversion to lactate) (filled rectangle). D) Energy map of AT-MSCs and UC-MSCs with OCR vs ECAR of AT- (blue spheres) and UC-MSCs (orange spheres) are plotted. E) The plot shows the ratio between the mitochondrial ATP production rate and the glycolytic ATP production rate (logarithmic scale). Data expressed as points (A, B, D, E plots) and column chart (C plot) represent the mean ± SD (vertical bars) from three experiments carried out on different cell preparations. * indicates significant differences (P<0.05).

The O_2_ consumption rate (OCR) and the extracellular acidification rate (ECAR) by lactate production were simultaneously measured in AT-MSCs (Fig 5A) and UC-MSCs under basal conditions (Fig 5B). In order to detect the total ATP production rates in living MSCs, serial additions of oligomycin (olig), a specific inhibitor of the mitochondrial ATP synthase, and of rotenone plus antimycin A (Rot+AA), inhibitors of mitochondrial complex I and III, respectively, were automatically and stepwise performed. This metabolic assay carried out according to Seahorse XFp technology, allowed us to evaluate the amount of ATP produced by OXPHOS and glycolysis, which represent the two main metabolic pathways responsible for ATP production in mammalian cells. The plots of Fig 5A and 5B highlight the OCR decrease due to the ADP phosphorylation inhibition by oligomycin and the block of electron transport chain by Rot+AA coupled to an increase in ECAR, which mirrors glycolytic rate. From the flux of O_2_ consumption and H^+^ production, the ATP production rates by OXPHOS and glycolysis were calculated (Fig 5C). AT- and UC-MSCs showed a different total ATP production rate, since OXPHOS and glycolytic pathways in UC-MSCs provided a higher amount (P<0.05) of cellular ATP than in AT-MSCs (Fig 5C). The energy map of both MSC types corroborates an aerobic energy metabolism with more active OXPHOS and glycolytic pathways in UC-MSCs than AT-MSCs (Fig 5D) (P<0.05). However, AT-MSCs showed a higher mitoATP/glycoATP ratio than UC-MSCs, which highlights a prevailing oxidative phenotype (Fig 5E) (P<0.05).

### Mitochondrial respiration

**Fig 6:**
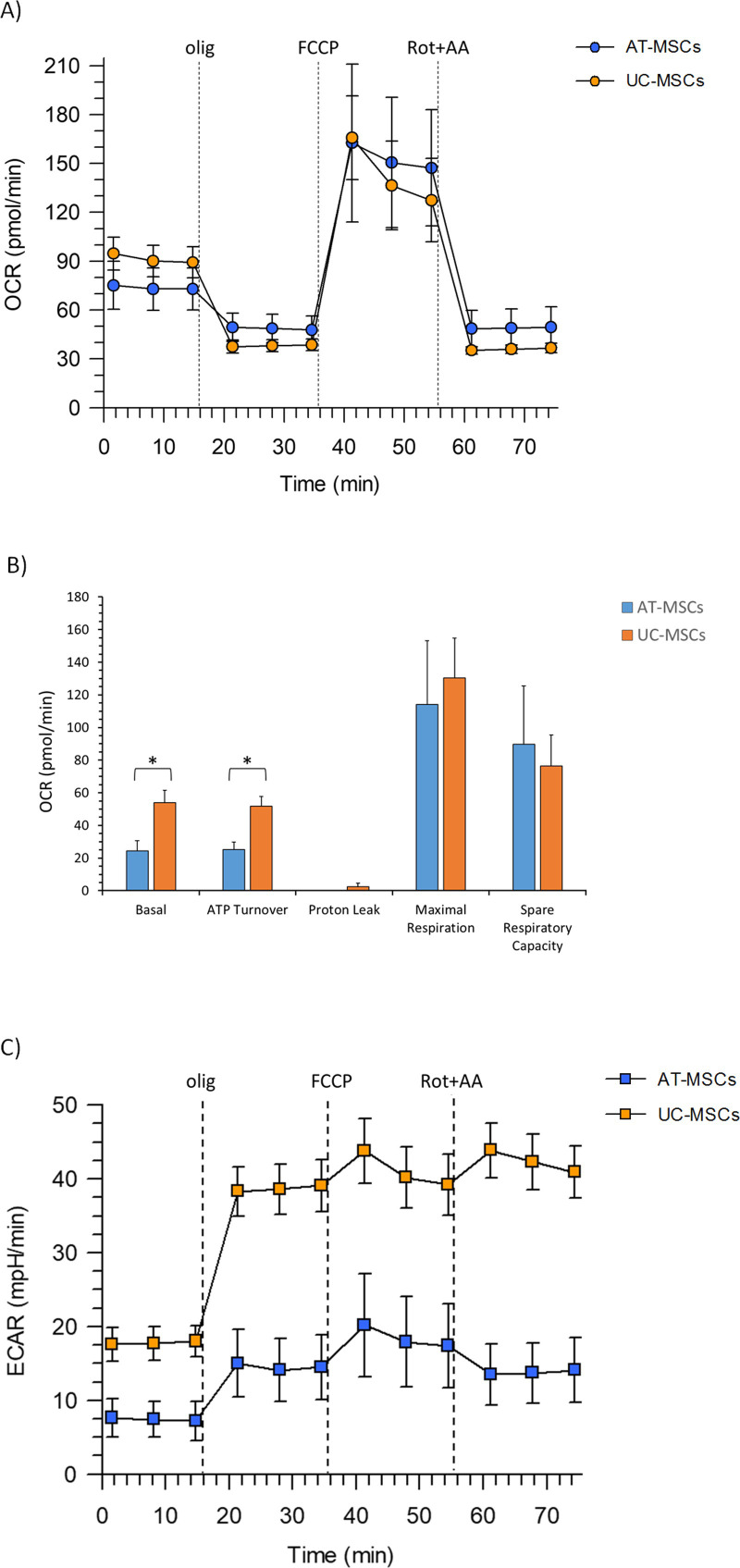
Metabolic measurements in MSCs. A) Mitochondrial respiration profile (OCR) of AT-and UC-MSCs under basal conditions and after the addition of 1.5 μM oligomycin, 2.0 μM and 0.5 μM FCCP and a mixture of rotenone plus antimycin A (0.5 μM) injections where indicated by the dotted lines. B) Mitochondrial parameters (basal respiration, ATP production, proton leak, maximal respiration, spare respiratory capacity) in AT- and UC-MSCs. C) Basal ECAR and ECAR changes after addition of the mitochondrial inhibitors: 1.5 μM oligomycin, 2.0 μM and 0.5 μM FCCP and a mixture of rotenone plus antimycin A (0.5 μM) addition on AT- and UC-MSCs. Data expressed as points (A, C) and column chart (B) represent the mean ± SD (vertical bars) from three experiments carried out on different cell preparations. * indicates significant differences (P<0.05).

The key parameters of mitochondrial function, directly measured by the cell respiration profile of MSCs, are shown in Fig 6A. Both basal respiration and ATP turnover show significantly higher values in UC-MSCs than in AT-MSCs (Fig 6B) (P<0.05). Noteworthy, oligomycin inhibition completely blocks the mitochondrial oxygen consumption without any proton leak detection. The same OCR values of basal respiration and ATP turnover confirm an almost 100% coupling efficiency. The cellular ATP demand in MSCs, when OXPHOS is inhibited by oligomycin, is fulfilled by the activation of glycolytic pathway (Fig 6C). AT- and UC-MSCs increase the maximal respiration by four and two orders of magnitude, respectively after FCCP addition. Consequently, the spare respiratory capacity, currently taken as a parameter which defines the cell propensity to differentiate [32, 33], represents 79% and 59% maximal respiration for AT- and UC-MSCs, respectively.

## Discussion

In the present study, MSCs from canine umbilical cord and adipose tissue were isolated and several features were evaluated: *in vitro* proliferation, migration ability, spheroid formation capacity, differentiation potential, expression of stem markers and, for the first time in the dog, cell bioenergetics. Usually, for clinical application, cells are used during the first *in vitro* passages, to avoid any side effects due to senescence and cell transformation following a long culture period. Thus, only the first four culture passages were considered in this study. UC samples were collected from fetuses of a single female dog, so there could be a lack of individual variation for the fetal-adnexa derived MSCs, while AT sample were obtained from different animals, so the individual variation exists, but the animals were all young, therefore age influence should be minimal. Nevertheless, it is likely that in the present study there was more variability for adult derived MSCs than for fetal adnexa derived ones.

Cell proliferation was similar for canine AT- and UC-MSCs, in accordance with the results observed in our laboratory for horse MSCs using the same protocol for DT calculation [34], but in contrast with data obtained in the dog [19], where AT-MSCs had a lower DT than UC-MSCs. The DTs for both MSCs lineages reported in that research [19] were shorter than those observed in the present study. These differences can be ascribed to different factors or to their combination, such as the different cell growth curve production method used to calculate DT, the different culture conditions, and also the different variability of MSC sources, since tissues where collected from the same animals. Indeed, when the same DT calculation method adopted in our research was applied, DTs of canine UC-MSCs from other studies [15, 35–37] were similar to our present results.

As previously observed [10], canine AT-MSCs presented a progressive increase in DT length over time, but with a slightly different trend for UC-MSCs. In fact, UC-MSCs showed a longer DT at first passage after thawing, and a progressive increase in DT over time starting from the second passage. This behaviour, never observed in other studies using non cryopreserved canine UC-MSCs [15, 35], may be explained as a freezing-thawing consequence, and could be related to a higher UC-MSCs sensitivity to the cryopreservation protocol used.

Colony formation was similar in both canine cell cultures. However, the mean number of colonies obtained in this study for AT and UC-MSCs is lower when compared with what found for canine amniotic membrane derived MSCs using the same protocol [23]. It is likely that different cells and culture medium influenced the results.

Migration potential of MSCs, also known as wound healing ability, is considered an important feature for their integration into the host tissue during therapeutic applications [38]. In the present study, the migration ability of canine AT- and UC-MSCs were similar. Nevertheless, the migration capacity of AT-MSCs observed in the present study was lower than that previously obtained [10], probably due to an individual variability and a different culture medium used.

Self-assembly of MSCs into aggregates has significant implication in their applications in cell therapy and tissue regeneration [39]. For example, the 3D spheroid culture system contributes to an optimization for efficient *in vitro* differentiation of MSCs [40], and *in vivo* MSC 3D transplantation of synovial MSC aggregates promoted cartilage tissue regeneration in a rabbit model [41]. Therefore, since the incidence in several breeds of osteoarticular pathologies in the dog, such as dysplasia or arthrosis, and the possibility of improving cellular treatments, the canine MSCs ability to form spheroids was investigated. This is the first report evaluating canine adult and fetal adnexa MSCs ability to form spheroids *in vitro*, and for this purpose, a specific multi-well plate was used. In this culture conditions, both cell cultures were able to form spheroids of similar volume, differently from what observed between adult and fetal adnexa derived cells in the horse [34]. This finding for canine MSC lineages is then supported by their similar ability to differentiate toward chondrogenic lineage. Accordingly, the *in vitro* MSC self-assembly as 3D aggregates has been suggested to recapitulate the *in vivo* mesenchymal condensation events that influence MSC properties beyond chondrogenic lineage [39]. Indeed, no difference was also observed in the differentiation ability of the studied cultured cells, in neither adipogenic nor osteogenic sense, confirming what already demonstrated for canine MSCs from different tissues [19].

On considering the molecular characteristics of studied cells, both lineages were positive for the mesenchymal markers CD90 and CD44, and negative for the hematopoietic marker CD14, as postulated by the International Society for Cellular Therapy [2]. On the other hand, differently from Zhan et al. [19], we observed the expression of CD34 by canine AT-MSCs, while UC-MSCs expressed this marker only at P0, as also recently observed for canine MSCs from UC perivascular tissue [42]. Nonetheless, nowadays the negative expression of CD34 is not considered an essential feature of MSCs, because it was demonstrated that the expression of this marker could be influenced by the environment, the cellular type and by the passage of *in vitro* culture [43]. As previously demonstrated [35, 44], a weak expression of DLA-DRA1 and DLA-DQA1 markers was observed in UC-MSCs only at passage 0. The weak detection of these markers can be attributed to contamination with other cells present in the cord matrix (endothelial cells, blood cells, etc.). Conversely, in the present study all AT-MSCs showed a weak expression of the above markers in almost all the passages. These data confirm that also in the dog mesenchymal stem cells isolated from the umbilical cord would seem less immunogenic and therefore safer for heterologous transplantation compared with adipose tissue derived counterparts [45].

The results show that both AT- and UC-MSCs mainly rely on OXPHOS to produce ATP, but they can increase the anaerobic glycolytic pathway when the mitochondrial respiration is inhibited to fulfil the ATP demand. Therefore, the elevated dependence on the glycolytic flux, which is a common metabolic feature of stem and cancer cells, is maintained even if temporarily latent and may also represent an environmental adaptation strategy to maintain stem cell identity. Even if MSCs are the most commonly tested stem cells in experimental cell therapy [46–48], the mechanisms underpinning phenotypic and functional properties, which underlie the bioenergetic changes during differentiation, are not well understood. Apparently, the MSC metabolic plasticity and versatility respond to the cellular demands and the related energy requirements [46, 47]. Therefore, MSC fate in animal species can be addressed by energy metabolism, which in turn can be affected by many variables such as stemness [48], culture conditions [47] and even the individual physiological state [49]. In general, proliferating stem cells do not use OXPHOS [47], even if they maintain the ability to shift to OXPHOS when a more efficient ATP production is required, as shown by the results. Accordingly, mitochondrial dynamics significantly impacts cell function and fate [50] and metabolic reconfiguration is strictly associated to a different mitochondrial function [48]. Since stem cells can drive the plasticity in energy metabolism to satisfy their proliferative, differentiation and reprogramming state [51], we found that the canine MSCs studied not necessarily use the energy-consuming anabolic metabolism to synthesize the building blocks for macromolecules and biostructures [52], but they prefer to mainly exploit substrate catabolism to produce ATP. Although UC-MSCs showed higher ATP production than AT-MSCs, the latter synthesize more ATP by mitochondrial activity. However, in both cell cultures mitochondria have a high coupling efficiency, namely they efficiently match electron transport to ADP phosphorylation to yield ATP. To summarize, these cells are both able to address the whole energy obtained from substrate oxidation to ATP production, a feature found in non-specialized cell cultures. Importantly, AT- and UC-MSCs do not work at the limit of their bioenergetic capacity. They possess a spare respiratory capacity that exceeds by several orders of magnitude their basal OCR. This feature can guarantee the increase in aerobic metabolism required to satisfy the increased ATP demand during cell differentiation [53].

## Conclusions

In mammalian cells, the cellular ATP is provided by OXPHOS and glycolytic pathways. The proportion between these two distinct metabolic strategies mainly depends on the cell type, the physiological state and the related energy requirements. The two types of canine MSCs under study apparently share the same bioenergetic strategy, namely a OXPHOS prevalence, and possess other shared biological features, even if some differences exist in the expression of some molecular markers, such as CD34 and DLA-DQA1, and bioenergetic parameters. Further studies will help to elucidate the impact of cell passages and of the individual variability on cell metabolism and bioenergetics. The integration between histological, biological and bioenergetic studies can depict an innovative pattern of the cellular phenotype and of the metabolic strategies required to modulate control of stem cell progression and differentiation. Accordingly, the information obtained may efficiently contribute to select the most appropriate MSCs, culture and experimental conditions to carry out transplantation experiments in mesenchymal stem cell therapy for companion animals and strengthen the emerging hopes in regenerative and preventive medicine. The present data indicate that, between the two canine MSC types under study, most likely, due to the lower immunogenic properties and higher efficiency of ATP production, UC-MSCs may be more appropriate for transplantation studies, even if studies on the biological and metabolic properties of canine MSCs are still at the beginning. Undoubtedly, both MSC types, easy to obtain and endowed with an intriguing metabolic versatility, represent a very interesting matter of investigation and a putative potential tool in regenerative medicine. Present data not only stimulate further studies to substantiate this intriguing hypothesis, but also strengthen the relevance of an increased knowledge of stem cell bioenergetics to address actions to improve the quality of *in vitro* cultures. Accordingly, the knowledge of the mitochondrial status and especially of some bioenergetics parameters such as the spare respiratory capacity, which guarantees a metabolic flexibility, may help to select the better candidates for transplantation studies [54].

## Supporting information

S1 Raw images (PDF)
