# Supplementary figures and images for: Biological characteristics and metabolic profile of canine mesenchymal stem cells isolated from adipose tissue and umbilical cord matrix
Source: PLoS One. 2021 Mar 4;16(3):e0247567. doi: 10.1371/journal.pone.0247567 (PMC7932077; doi:10.1371/journal.pone.0247567)

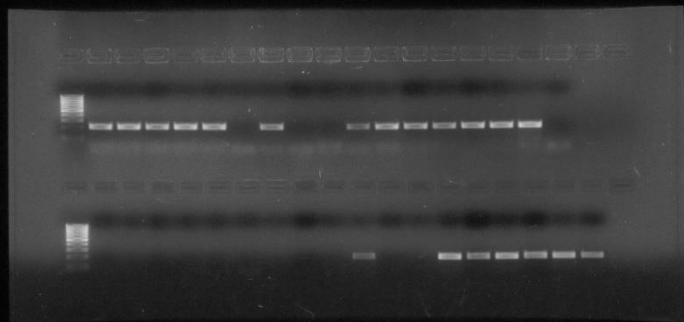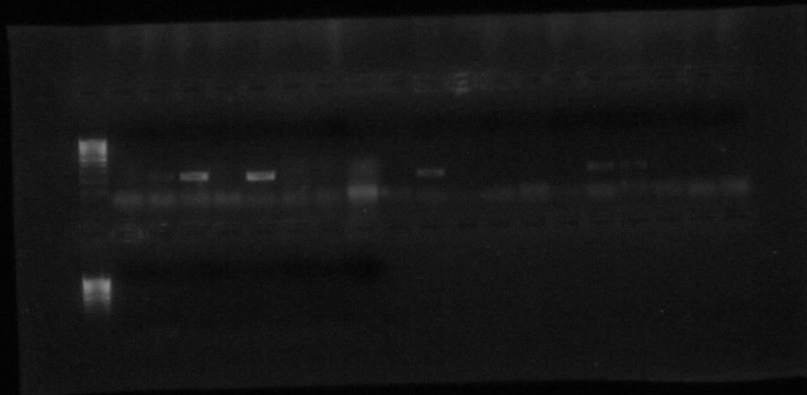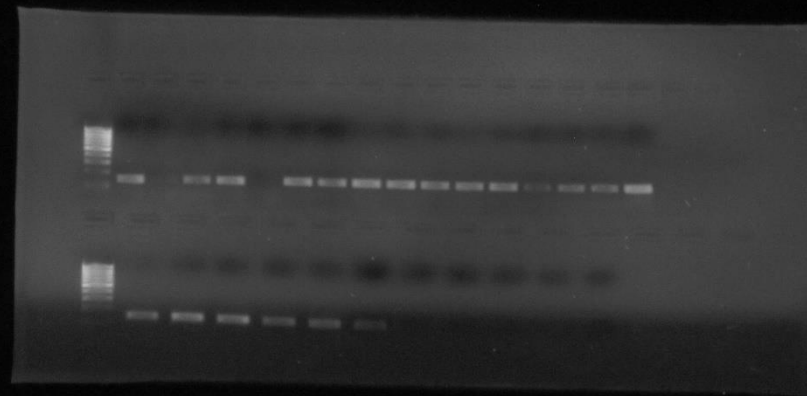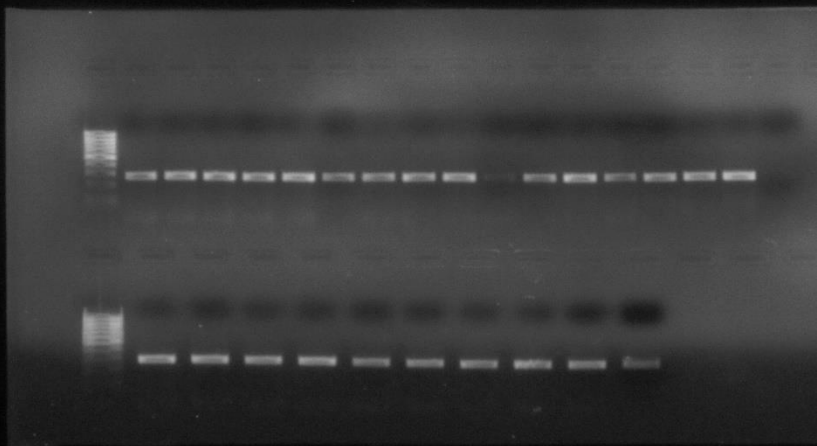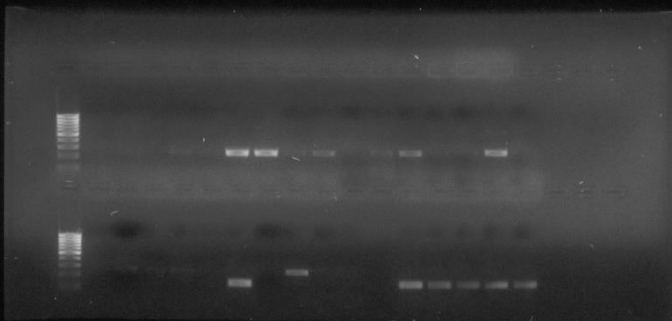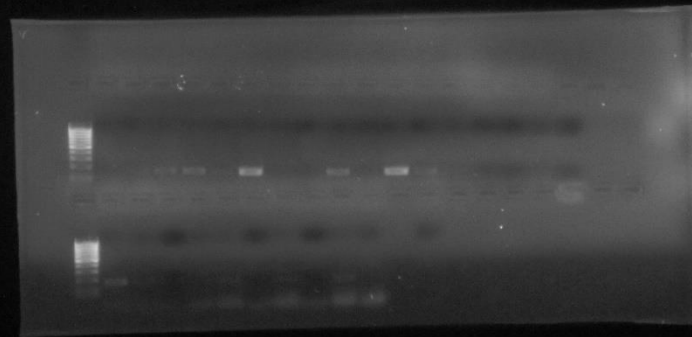

Supplement: S1 Raw images — (PDF) [file pone.0247567.s001.pdf]
